# P-1383. Gap in Doxy PEP Prescription for People Living With HIV Compared with PrEP Users in a Primary Care Practice

**DOI:** 10.1093/ofid/ofae631.1559

**Published:** 2025-01-29

**Authors:** Elizabeth Asiago-Reddy, Nabeil Himat, Courtney Myers

**Affiliations:** SUNY Upstate Medical University, Syracuse, New York; SUNY Upstate Medical University, Syracuse, New York; SUNY Upstate Medical University, Syracuse, New York

## Abstract

**Background:**

Use of 200mg of doxycycline as post-exposure prophylaxis within 72 hours of a condomless sexual encounter (Doxy PEP) has been shown to reduce the risk of key sexually transmitted infections (STIs). Doxy PEP is effective both for people living with HIV (PLH) as well as those who do not have HIV. The goal of this analysis was to describe uptake of Doxy PEP in a primary care practice in Syracuse, NY, a Northeastern U.S. city serving a large population of PLH as well as people prescribed pre-exposure prophylaxis (PrEP) for HIV prevention.

**Methods:**

Data were abstracted from the electronic medical record. Patients who were seen for HIV or PrEP care between 1 Jan and 31 December, 2023 who were transgender women (TGW) or cisgender men self-identified as gay, bisexual, or “other” sexual orientation (MSM) and had been diagnosed with chlamydia, gonorrhea or syphilis during 2023 were analyzed. Doxy PEP discussion and prescription were determined through medication review and office notes up through the first quarter of 2024. Factors associated with providers recommending and/or prescribing doxy PEP were evaluated using bivariable and multivariate logistic regression.

**Results:**

A total of 602 MSM and 25 TGW were seen in the practice during 2023; 117 (18.7%) were diagnosed with at least one STI, specifically syphilis [37; (31.6%)], chlamydia [33 (28.2%)], gonorrhea [23 (19.7%)] or more than 1 of these [24 (20.5%)]. A total of 46 (39.3%) patients were prescribed doxy PEP; 11 (9.3%) declined, and 60 (51.3%) had no documented discussion or prescription. Most 35 (76.1%) prescriptions were in the 1st quarter of 2024; average days between STI diagnosis and doxy PEP prescription was 200 days. On multivariate analysis (MVA), only PrEP vs. HIV was associated with prescription (OR 13.3 p< 0.0001; this held even after excluding 4^th^ quarter STI diagnoses to account for less frequent follow up of PLH vs. PrEP users (OR 17.5, p=0.001). Doxy PEP prescription was not associated with age, race or ethnicity, type or site of STI, provider, or time (days) since STI diagnosis.

**Conclusion:**

Doxy PEP is a valuable tool for reducing STIs; it may be underutilized in people living with HIV despite ongoing risk, and methods to encourage prescription for PLH are warranted.

**Disclosures:**

**Elizabeth Asiago-Reddy, MD**, Abbvie: Grant/Research Support|GSK/Viiv: Grant/Research Support|Pfizer: Grant/Research Support|Theratechnologies, Inc.: Grant/Research Support

